# The Wet Blanket Pack in Dysmenorrhea, Neuralgia, Etc.

**Published:** 1877-07-20

**Authors:** B. H. Washington

**Affiliations:** Augusta, Georgia


### THE WET BLANKET PACK IN DYSMENORRHEA, NEURALGIA, &c.

By B. H. WASHINGTON, M. D., Augusta, Georgia.

Having used water very extensively in my practice it may prove profitable to younger member of the profession to report some remarkably successful cases. We quickly abandoned the wet sheet pack, for it was too troublesome to use in acute febrile cases for the purpose of cooling the fever down; wet towels from clavicle to pubis, changed as often as they get warm, being far more convenient and efficacious. In chronic cases the cold wet sheet pack was too danger, ous to confide to domestic management-for a severe or fatal congestion might result at any time. We, therefore, abandoned the cold wet sheet pack, and in all chronic cases substituted the hot wet blanket pack, and oftentimes with surprising succe s. The bed clothes should be spread on the bed, as many as may be sufficient to keep the patient perfectly comfortable; the blanket should be wrung out of water as hot as the hands can bear, and then spread on the bed, and the patient, stripped, be placed thereon as soon as the blanket is cool enough to allow its use; the bed clothes should then be carefully tucked so as to exclude the entrance of any air beneath, or the formation of any large air chamber beneath the covering, as in either case the patient will be very uncomfortable, and but little good will be accomplished. The patient should remain in the pack at least one hour and a half, and if comfortable, he might remain in four or five hours, or even all night, if quiet; in the latter case some one should sit up so as to prevent the patient from throwing off the cover and incurring danger thereby.

The blanket pack should be used ordinarily about every alternate night; though in many cases, if used as an anodyne, it might be used every day; if used too often the skin may be stimulated too highly and its action be rendered very irregular, sometimes stopping and starting again, causing a feverish pulse perhaps half a dozen times a day. When the patient is first placed in the pack it is very agreeable, but in the course of eight or ten minutes the body abstracts so much heat from the blanket a slight coolness is felt, but that soon wears off; if, however, the patient complains of feeling cool and uncomfortable all the time he is in the pack, it is a sure indication that the nervous system is considerably out of order. To prevent the patient from being so sensitive to cold, it will be advisable to use friction on the skin freely, and bromide of potassa or quinine internally.

The patient should be rubbed dry with warm towels, and should not be put in the pack in a cold room. To show the extraordinary benefit derivable from the blanket pack, the following cases are reported:

*Dysmenorrhea.*—Mrs.——, aged about thirty-five, Augusta. Called in consultation with my son; found the patient had been tainted with syphilis by her husband, and afflicted terribly for fourteen years. In addition she had been afflicted with dysmenorrhea for ten years of that time, and neuralgic pains very irregular indeed, sometimes coming on in the back, head or uterine region, every two, three, or four weeks. At her regular monthly period, the pains, most commonly in her head, were intolerable, and for some years had frequently been so severe as to bring on epileptic convulsions. During the fourteen years twenty-eight physicians had exhausted their skill on both diseases without any success. She was in good spirits in regard to her syphilitic ulcerations, for she had improved very remarkably under my son's treatment, but the headache at her monthly periods still caused the most intense agony. My son, quite familiar with my dry cupping practice, had tried it, but though some improvement was effected, yet the dysmenorrheal pains might be fairly said to have defied him as they had done others. The diagnosis was that it was a case of neuralgic dysmenorrhea; we, therefore, recommended the continuation of the dry cupping, and, in addition, the wet blanket pack for at least one hour and a half every alternate day, and longer if she could sleep comfortably in it.

The treatment was faithfully kept up for two months, and at the end of that time her monthly period passed without pain, and for five or six months she continued as regular and natural as—in her own language—any healthy young girl. Latterly the pains began to return. She has been put under the same treatment, and she is now nearly entirely relieved again. When we compare the above success with the want of success of the twenty eight physicians who had tried the case previously, and with the following from Barnes on Diseases of Women, no one can hesitate for a moment in deciding which is the best plan of treatment of that form of dysmenorrhea. Barnes says, p. 196:

"The obstinate character of the affection is well known. It may be predicted with some confidence that a girl who starts with dysmenorrhea is doomed to suffer for years, perhaps for life. It is said sometimes to wear its self out; occasionally marriage, if fruitful, brings relief; but more frequently the recurring attacks of pain, even if unattended by other causes of distress, increase the irritability of the nervous centres, impair nutrition, destroy the harmony or correlation of the vital forces, and reduce the sufferer to the condition of a perpetual invalid, enjoying, at best, only a comparative remission of illness. If pains do not persist throughout the intermenstrual intervals, it is liable to be evoked by any fatigue, or emotion, so that the state of the patient comes to be the chief care of the household."—*Nash. Jour. Med.*

[Dr. Washington reports cases of neuralgia, severe cold, cramps in the colapse of cholera and acute pericarditis relieved by this method.]

